# Long-term follow-up of laparoscopic treatment and hepaticojejunostomy without Roux-en-Y for choledochal cyst in children. Report of two cases and review of the literature

**DOI:** 10.1016/j.ijscr.2025.111151

**Published:** 2025-03-14

**Authors:** Antonio Gonçalves de Oliveira-Filho, Maria Giovana Oliveira Farias, Márcia Alessandra Cavalaro Pereira da Silva, Ana Paula Nunes Bento, Joaquim Murray Bustorff-Silva

**Affiliations:** Department of Pediatric Surgery, School of Medical Sciences of Unicamp – University of Campinas, Rua Tessalia Vieira de Camargo, 126, CEP 13.083-887 Campinas, São Paulo State, Brazil

**Keywords:** Hepaticojejunostomy, Hepaticoduodenostomy, Laparoscopy, Choledochal cyst, Case report

## Abstract

**Introduction and importance:**

Complete removal of the choledochal cysts (CC) with biliary enteric reconstruction is the standard treatment. The preferred biliary reconstruction, by either hepaticoduodenostomy (HD) or Roux-en-Y hepaticojejunostomy (RYHJ) remains a personal choice according to the surgeon's preference and experience.

We report our long-term follow-up using an alternative method of biliary-enteric anastomosis without Roux-en-Y.

**Case presentation:**

Two children (1½ and 4½ years old) with type I CC, diagnosed by CT scans or MRI in 2012 and 2013, underwent complete resection of the cysts and an end-to-side hepaticojejunostomy without Roux-en-Y (HJWRY) totally by laparoscopy. Bile leakage has occurred but, resolved spontaneously and oral feeding could be resumed. The children were discharged on the 6th and 8th postoperative days.

At a 12-year follow-up, both children were doing well without complaints, and have shown no episodes of cholangitis. At the last follow-up, laboratory tests and ultrasound examinations were normal.

**Clinical discussion:**

The main treatment of choledochal cysts is their complete resection with biliary enteric reconstruction, which intends to mitigate the risk of malignancy and prevent postoperative cholangitis. The choice of enteric biliary reconstruction is still a matter of debate between HD and RYHJ, and this alternative HJWRY can be another strategy to compose the therapeutic options for the surgeon.

**Conclusion:**

Laparoscopic resection of choledochal cysts in children with the alternative HJWRY, appears to be a safe, simple, and reliable technique, does not facilitate reflux of bile into the stomach, has only one anastomosis, and could be considered a more physiological operation.

## Highlights

• Alternative biliary-enteric reconstruction for treatment of choledochal cysts
• Long-term follow-up (12 years) without any complications.
• Just one anastomosis
• No reflux of bile into the stomach.

## 1 Introduction

A choledochal cyst (CC) is a rare congenital anomaly characterized by a cystic dilatation of the bile ducts. If left untreated, complications such as recurrent acute cholangitis, pancreatitis, and malignant transformation into cholangiocarcinoma may occur. (1,2).

Complete removal of the cyst with biliary enteric reconstruction is the standard treatment for CC. However, the choice of approach, either by laparotomy or laparoscopy, and the type of biliary-enteric anastomosis are still controversial. ([2], [3], [4]).

Although laparoscopy is currently the preferred approach, biliary reconstruction by either hepaticoduodenostomy (HD) or Roux-en-Y hepaticojejunostomy (RYHJ) remains a personal choice according to the surgeon's preference and experience (3,4).

We report our experience and the long-term follow-up of two children with choledochal cysts, treated by laparoscopic cystic resection and biliary-enteric reconstruction with an end-to-side hepaticojejunostomy without Roux-en-Y.

This manuscript was prepared following the SCARE guidelines to ensure a structured and comprehensive presentation of the case (5).

## 2 Case report

### 2.1 Case 1

A 4½-year-old girl was treated at the municipal hospital for cholangitis in August 2012. Ultrasound and computed tomography showed a 4 × 3.4 cm bile duct cyst with a volume of 38 cm3 at that time. A magnetic resonance cholangiopancreatography performed in September 2012 showed a fusiform choledochal dilatation with a diameter of 3 cm. She was referred to our hospital with a diagnosis of a Todani I-A choledochal cyst.

The child underwent surgery on October, 2012.

**Fig. 1:**
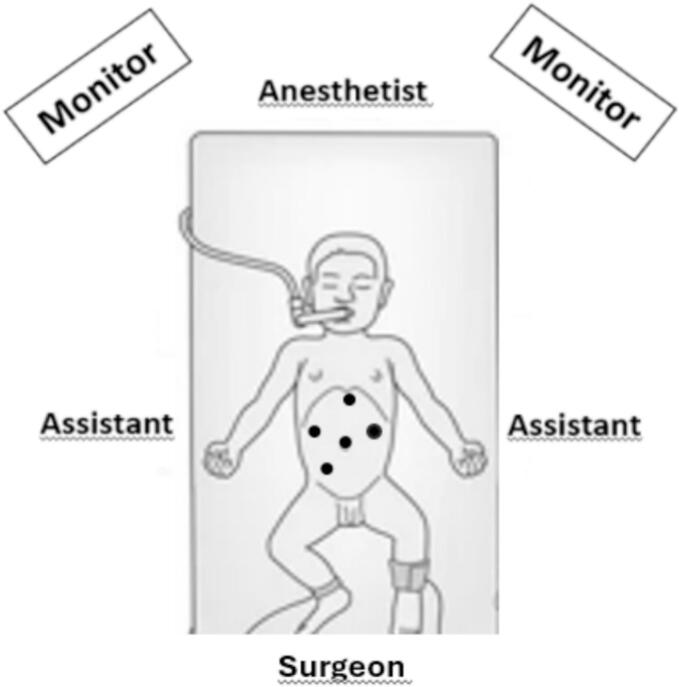
Team position, and trocar insertions.

Under general anesthesia, the child was positioned according to the French position for laparoscopic surgery, but without elevation of the legs (Fig. 1).

A 10 mmHg pneumoperitoneum using CO2 was achieved through a Veress needle at the umbilicus and five trocars were placed: four 5-mm trocars: at the umbilicus, next to the xiphoid appendix, in the right upper and lower quadrants and one 10-mm trocar in the left upper quadrant to accommodate hooks, needle holder, gauze, etc. (Fig. 1).

The choledochal cyst was identified and fully dissected, separated gently from the portal vein and hepatic artery, and then ligated distally close to the duodenum. The gallbladder was detached from the liver, the cystic duct was followed to the choledochal cyst, and the common hepatic duct was identified and transected close to the confluence of the left and right hepatic ducts. The specimen (choledochal cyst and gallbladder) was removed through the 10 mm trocar.

The Treitz ligament was identified, and a small incision was made with the cautery at the antimesenteric edge of the most proximal part of the jejunum that could reach the hepatic hilum without tension (about 15–20 cm from the Treitz ligament).

**Fig. 2:**
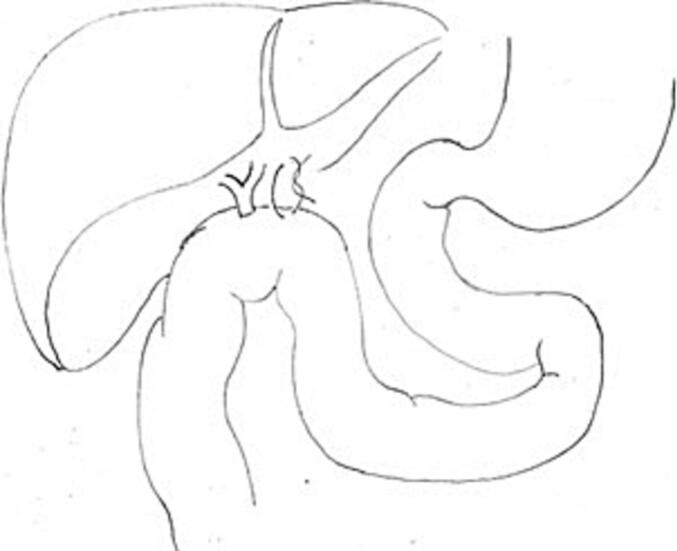
End-to-side hepaticojejunostomy without Roux-en-Y.

The intracorporeal laparoscopic anastomosis was created between the common hepatic duct and the jejunum with a 5–0 Vycril® running suture at its posterior edge and interrupted sutures at its anterior edge. To avoid torsion, an additional stitch was placed to fix the intestinal loop near the round ligament fissure (Fig. 2).

A Penrose drain was left in place near the anastomosis and exteriorized through the right upper quadrant port incision. The pneumoperitoneum was released, and the incisions closed.

The immediate postoperative period was uneventful, but bile leakage through the Penrose drain was noted on the second day, with a total volume of 188 ml in 24 h. The drainage persisted until postoperative day 4 when oral feeding was resumed. Bile leakage stopped on the 5th day (Clavier-Dindo I). The patient was started on a full diet and was discharged on postoperative day 6.

The child hasn't had any complications during this long post-operatory period, and at the last follow-up examination (12 years after the surgery), the child was in good health, without any complaints, and with normal laboratory values. The ultrasound showed a normal liver, without dilatation of the bile duct or stones, but with minimal aerobilia.

### 2.2 Case 2

A 1 1/2 year-old little girl complained of abdominal pain and a palpable mass in the right upper quadrant of the abdomen. Neither jaundice nor hypocolic stools were noted.

Computed tomography performed in January 2013 showed a cyst near the hepatic hilus measuring 5.5 × 3.9 × 3.5 cm with a volume of 39 cm3.

The diagnosis was choledochal cyst I-A according to the Todani classification.

The child was operated on March, 2013.

Under general anesthesia, the child was placed in the frog leg position at the end of the operating table. The team was also positioned according to the French position in this case. The same technique was used as in case 1 and the operation proceeded without complications.

The immediate postoperative period was uneventful, but the bile leakage through the Penrose drain was noted on the first postoperative day, with a total volume of 160 ml in 24 h. The bile leak stopped on postoperative day 6 and the oral diet was resumed (Clavier-Dindo I).

The child was discharged on postoperative day 8 with full oral feeding.

She is under continuous outpatient follow-up (12 years) and has no complaints or complications. Laboratory tests were always normal, and ultrasound always showed a normal liver with no bile duct dilatation, neither air nor calculi inside.

## 3 Discussion

We report here, to the best of our knowledge, an alternative type of bilio-enteric reconstruction after resection of a choledochal cyst, which has never been used in this disease before. These two girls, diagnosed with a type I choledochal cyst, underwent laparoscopic surgery with resection of the choledochal cyst and a hepatic-jejunal anastomosis without Roux-en-Y (HJWRY) to treat their condition. Both operations were performed entirely laparoscopically, without intraoperative complications, as described above. Both operations have been done by the same pediatric surgeon.

This technique proved to be simple and safe and could be performed entirely by laparoscopy, with obvious advantages over the two commonly performed techniques (HD or RYHJ). Hepaticojejunostomy reconstruction without Roux-en-Y, performed without transection of the jejunum, is a more physiologic technique than reconstruction with Roux-en-Y, as the integrity of the jejunum is preserved, allowing normal conduction of myenteric impulses and thus not interfering with normal bowel movements.

Treatment of choledochal cysts in children always requires complete resection of the cyst through an open, laparoscopic, or robotic approach, but bilio-enteric reconstruction is preferably performed either by a Roux-en-Y hepaticojejunostomy (RYHJ) or a hepaticoduodenostomy (HD), depending on the surgeon's choice and experience (4,6).

Other techniques such as a modified biliary-enteric anastomosis for choledochal cysts (2), interposition of a jejunal segment (with or without a valve) (7,8) or an appendix ([9], [10], [11], [12]) between the hepatic duct and duodenum have been described but are not commonly used. The gradual introduction of laparoscopic techniques to treat this entity has prompted pediatric surgeons to look for a technique involving fewer anastomoses.

Several studies have shown that both HD and RYHJ are suitable for restoring bilio-enteric continuity after resection of the choledochal cyst. However, there are some differences between the two methods that need to be considered. One advantage of HD over RYHJ is the simplicity of the procedure, with fewer anastomosis, which results in significantly shorter operating times and less surgical bleeding. In addition, patients who undergo HD tend to have a shorter hospital stay than patients who undergo RYHJ. On the other hand, some have considered the proximity of hepatoenterostomy to the stomach as a possible risk factor for increased episodes of reflux/gastritis and cholangitis. In contrast, although involving a larger number of anastomoses, RYHJ is associated with a lower risk of these complications due to its more distal location. It is worth noting that there are no randomized controlled trials specifically comparing HD and HJ (4,6).

The traditional Roux-en-Y hepaticojejunostomy involves anastomosis of the remaining hepatic duct to a loop of jejunum to be excluded near its closed end (suture line #1), a jejuno-jejuno anastomosis (suture line# 2) to reconstruct the digestive transit and a third anastomosis to reconstruct the bilio-enteric flow (suture line# 3). The entire procedure can be completed laparoscopically, or the jejunum can be brought to the umbilical incision, and the Roux-en-Y constructed outside the abdominal cavity. The surgeon then reinserts the bowel into the abdominal cavity and performs the hepaticojejunostomy laparoscopically. The main advantage of this video-assisted technique is that it allows a safer extracorporeal anastomosis, although the hepaticojejunostomy is always performed by laparoscopy, which is associated with difficulties and risks of complications (13).

Open or laparoscopic resection of the choledochal cyst with Roux-en-Y reconstruction is a complex procedure that requires longer operating time and a higher level of technical expertise, but it is the most performed operation for treating the disease (4,6,[13], [14], [15]).

Another type of reconstruction of the bile-enteric flow after cyst resection is hepaticoduodenostomy. This technique creates a new connection between the remaining hepatic duct and the duodenum. According to several authors, this type of bilio-enteric reconstruction can be performed laparoscopically or robotically and, depending on the surgeon's choice, also openly (13,16).

Several systematic reviews compare hepaticojejunostomy with Roux-en-Y with hepaticoduodenostomy for bilio-enteric reconstruction and both procedures are effective in reconstructing bilio-enteric flow after resection of choledochal cysts and reducing the risk of complications such as pancreatitis and malignancy (4,6).

Both techniques appear to be similar in terms of complications such as cholangitis, fistulas, stenosis, bleeding, and stone formation. Although it did not reach statistical significance, cholangitis was reported to be slightly more common in Roux-en-Y reconstruction in a meta-analysis (4,6).

Despite operative time being significantly shorter in HD, hospital stay, adhesive bowel obstruction and time to oral feeding appear to be the same as in RYHJ (4,6,14).

Due to the technical characteristics of hepaticoduodenostomy, a significant index of biliary reflux into the stomach has been reported, which is not the case with Roux-en-Y hepaticojejunostomy (4,6). Hamada et al., in 2017, reported duodenogastric regurgitation in 14 of 17 cases reconstructed by HD and the necessity to convert 7 of them to hepaticojejunostomy due to persistent abdominal symptoms in long-term follow-up (14).

A modified biliary-enteric anastomosis for bilio-enteric flow reconstruction was reported in 2017. Through a 5 cm oblique incision, the cyst and gallbladder were resected, and bilio-enteric flow was restored by bringing the jejunum about 25 cm distal to the Treitz ligament. An end-to-side anastomosis of the bile duct with the jejunum was created anterior to the transverse colon. The afferent loop of the jejunum was ligated with 1/0 silk suture and reinforced with interrupted seromuscular sutures 2 cm before the bile duct-enteric anastomosis. Finally, a lateral jejunojejunostomy was created between the afferent loop (about 10 cm after the Treitz ligament and the efferent loop about 25 cm from the bile duct anastomosis. They reported good initial results in 91 children. All operations were performed through an open approach (2).

Hepaticoduodenostomy is a simpler technique that is performed without transection of the small bowel and therefore does not interfere with normal peristaltic movements. In the post-meal period, the duodenal mucosa is exposed to rapid luminal pH changes from 2 to 7 due to the mixing of HCO3– with gastric acid from the stomach, but the duodenal mucosa has its physiologic defense mechanisms to prevent injury [17]. In the proximal jejunum, pH values of 7–8 prevail. So, there is a difference in intraluminal acidity in the part of the intestine that is anastomosed with the hepatic duct. If this might represent an advantage in terms of inflammatory processes at the hepatic duct after reconstruction, since the epithelium of the biliary tract has no physiological defenses, remains to be investigated. Just like in the hepaticoduodenostomy, the present technique allows direct contact of the intestinal contents with the hepatic duct, without a valve. According to a systematic review, HD does not appear to increase the incidence of cholangitis compared to HJ. In fact, more cases of cholangitis occur with RYHJ compared to HD, although not significantly (6). With the technique presented here, no cholangitis occurred during the 12-year follow-up of these two children.

The modified biliary-enteric anastomosis technique described by Chen et al. 2017 differs from the 3 bilioenteric reconstructions described above in several ways. Firstly, it was only performed via an open approach. Second, although transection of the jejunum can be omitted, which helps to maintain the integrity of the jejunum and allows normal transit of myenteric impulses, this modified technique involves ligation of the afferent loop with a silk suture and requires a jejuno-jejuno anastomosis to establish normal transit for bile and food, which resembles a Roux en Y. Although good results have been reported, this procedure appears to be a more complex operation, difficult to perform laparoscopically.

Our two cases developed bile leakage, which resolved spontaneously and were graded as Clavien–Dindo grade I (18,19).

Bile leakage is the most common postoperative complication reported after hepatobiliary surgery ranging from 3,0 to 20 % (20,21). Early biliary leakage in these two cases may be due to the type of suture between the jejuno and the hepatic duct. We have used a continuous suture on the posterior edge and an interrupted suture at the anterior edge that may have contributed to the leakage. Several authors recommend the use of continuous sutures in both anterior and posterior walls to reduce the chance of bile leakage, although with conflicting results ([22], [23], [24]).

## 4 Conclusion

Laparoscopic resection of choledochal cysts in children with the alternative hepatico-jejunostomy without Roux-en-Y anastomosis (HJWRY) appears to be a safe, simple and reliable technique with an uneventful long-term course. This procedure has advantages over hepatoduodenostomy, as it does not facilitate reflux of bile into the stomach, and over Roux-en-Y anastomosis, as it is a simpler procedure with only one anastomosis. Also, as it maintains intestinal continuity, it can be considered a more physiological operation. Further studies including a larger number of patients are needed to properly evaluate this technique's role in restoring bile-enteric flow after choledochal cyst excision.

## CRediT authorship contribution statement

**Antonio Gonçalves de Oliveira-Filho:** Writing – review & editing, Writing – original draft, Methodology, Formal analysis, Conceptualization, and Visualization.

**Maria Giovana Oliveira Farias:** Writing – review & editing, Conceptualization,

**Marcia Alessandra Cavalaro Pereira da Silva:** Writing – review & Editing.

**Ana Paula Nunes Bento:** Writing – review & Editing.

**Joaquim Murray Bustorff-Silva:** Writing – review & editing, Writing – original draft, Conceptualization and final validation.

## Consent

Written informed consent was obtained from the patient and parent for publication of this case report and accompanying images. A copy of the written consent is available for review by the Editor-in-Chief of this journal on request.

## Ethical approval

The study was reviewed and approved by the Research Ethics Committee following ethical guidelines.

## Guarantor

Antonio Gonçalves de Oliveira-Filho,MD, PhD.

## Sources of funding

None.

## Conflicts of interest

Authors have no conflict of interest to declare.
